# Improved Thyroid Hypoechogenicity Following Bariatric-Induced Weight Loss in Euthyroid Adults With Severe Obesity—a Pilot Study

**DOI:** 10.3389/fendo.2018.00488

**Published:** 2018-08-24

**Authors:** Ioannis Kyrou, Olu Adesanya, Nicholas Hedley, Sarah Wayte, Dimitris Grammatopoulos, Claire L. Thomas, Andrew Weedall, Subash Sivaraman, Lavanya Pelluri, Thomas M. Barber, Vinod Menon, Harpal S. Randeva, Miroslav Tedla, Martin O. Weickert

**Affiliations:** ^1^Aston Medical Research Institute, Aston Medical School, Aston University, Birmingham, United Kingdom; ^2^Warwickshire Institute for the Study of Diabetes, Endocrinology and Metabolism, University Hospitals Coventry and Warwickshire NHS Trust, Coventry, United Kingdom; ^3^Translational and Experimental Medicine, Division of Biomedical Sciences, Warwick Medical School, University of Warwick, Coventry, United Kingdom; ^4^Centre of Applied Biological and Exercise Sciences, Faculty of Health and Life Sciences, Coventry University, Coventry, United Kingdom; ^5^Department of Radiology, University Hospitals Coventry and Warwickshire NHS Trust, Coventry, United Kingdom; ^6^Radiology Physics Department, University Hospitals Coventry and Warwickshire NHS Trust, Coventry, United Kingdom; ^7^Department of Pathology, Institute of Precision Diagnostics and Translational Medicine, University Hospital Coventry and Warwickshire NHS Trust, Coventry, United Kingdom; ^8^Department of Clinical Biochemistry, University Hospital Coventry and Warwickshire NHS Trust, Coventry, United Kingdom; ^9^Department of Surgery, University Hospitals Coventry and Warwickshire NHS Trust, Coventry, United Kingdom; ^10^Department of ENT and Head and Neck Surgery, Faculty of Medicine, Comenius University, Bratislava, Slovakia

**Keywords:** obesity, bariatric surgery, ultrasound, thyroid echogenicity, gray-scale, TSH

## Abstract

**Background:** Obesity may affect both biochemical thyroid function tests; and thyroid morphology, as assessed using ultrasound scans (US). The aim of the present pilot study was to explore whether weight loss achieved by bariatric surgery alters thyroid US morphology including gray-scale measurements; and/or function in euthyroid adults with severe obesity.

**Methods:** Euthyroid adults (>18 years) with body mass index (BMI) ≥40 kg/m^2^ and negative thyroid peroxidase antibodies were assessed at baseline (pre-surgery) and after achieving at least 5% weight loss of their baseline body weight following bariatric surgery. Anthropometric assessments, biochemical/hormonal measurements (TSH, free-T4, free-T3, reverse-T3, and leptin) and thyroid US with gray-scale histogram analysis were performed at the baseline and post-surgery follow-up.

**Results:** Ten Caucasian, euthyroid patients (women/men: 8/2; age: 48.6 ± 3.1 years; BMI: 51.4 ± 1.8 kg/m^2^) successfully completed this study with significantly decreased body weight (>5% weight loss), waist circumference and serum leptin levels post-surgery (mean post-surgery follow-up duration: 16.5 ± 2.5 months). In parallel to the observed bariatric-induced weight loss, thyroid US echogenicity increased by 25% (*p* = 0.03), without significant changes in thyroid volume. No significant changes in thyroid function tests were detected. No significant correlations were observed between the increase in thyroid echogenicity and the decreases in anthropometric parameters and circulating leptin.

**Conclusion:** Our results indicate that in euthyroid adults with severe obesity, marked weight loss achieved by bariatric surgery is associated with a parallel significant increase in the thyroid US echogenicity, suggesting that morphological changes of the thyroid in obesity are reversible with weight loss.

**Clinical Trial Registration**: www.ClinicalTrials.gov, identifier: NCT03048708

## Introduction

By 2025, the global prevalence of obesity is expected to reach 18 and 21% in men and women, respectively, with a progressively increasing burden of obesity-related disease (1, 2). Thyroid function tests can be variably altered in patients with obesity (3–6). Whether such obesity-related changes of the thyroid function are a contributing cause, or a consequence of obesity is not always clear (3–6). Emerging evidence further suggests that the thyroid morphology can be also affected in obesity (7, 8). Indeed, a cross-sectional study in 186 overweight/obese children has documented that obesity is often associated with changes in the thyroid morphology, which can be identified by ultrasound scanning (US) and do not appear to be related to thyroid autoimmunity (e.g., thyroid hypo-echogenicity resembling, but not caused by autoimmune thyroiditis, AIT) (7).

Bariatric surgery remains the most effective treatment for obesity and is considered to induce a spectrum of metabolic/endocrine changes (9, 10). Despite the markedly increased number of patients treated by bariatric surgery in the past decade, only a few studies have investigated the potential effects of bariatric-induced weight loss on thyroid function (11, 12). To our knowledge, there are currently no studies on the potential changes in the morphology of the thyroid in patients with severe obesity after bariatric surgery. Thus, the aim of the present pilot study was to explore whether weight loss achieved by bariatric surgery may result in changes of the thyroid morphology and/or function in euthyroid adults with severe obesity.

## Methods and materials

### Study cohort

The Coventry/Warwickshire Research Ethics Committee approved this study (reference: 11/WM/0085), and all participants provided written informed consent in accordance with the Declaration of Helsinki. The study cohort was recruited from adults (>18 years) with body mass index (BMI) ≥40 kg/m^2^ (Class 3, severe obesity) and negative thyroid peroxidase antibodies (TPO-Abs), attending the obesity clinics at the University Hospitals Coventry and Warwickshire (UHCW) NHS Trust. The study exclusion criteria included known or newly detected thyroid disorders during screening, obesity secondary to primary endocrine/systemic disease (e.g., Cushing's syndrome) and any inflammatory disease (e.g., rheumatoid arthritis), as well as treatment with anti-inflammatory drugs (e.g., corticosteroids). For the purposes of this study, all recruited patients were prospectively followed at UHCW and were assessed at baseline (pre-surgery) and after achieving at least 5% weight loss of their baseline body weight following bariatric surgery. The latter was considered the minimum clinically relevant weight loss in the context of the present study.

### Anthropometric assessments

Anthropometric measurements were performed by trained staff at baseline and post-surgery follow-up, as per study protocol. Body weight (without shoes and heavy clothing) was measured to the nearest 0.5 kilogram (kg) using a digital platform scale suitable for patients with severe obesity (Seca, Germany). Waist circumference was measured to the nearest 1 cm with an inelastic measuring tape at the minimum circumference between the lower margin of the last rib and the superior iliac crest, with the participant in the upright position and at the end of expiration.

### Biochemical/hormonal analyses

Blood samples for biochemical assessments were obtained at the baseline and after post-surgery weight loss >5% was achieved. Samples were collected in the morning (8–10 am) after ≥10 h of overnight fasting. Serum/plasma samples were prepared and aliquoted immediately, and aliquots were stored at −80°C until assayed.

Routine biochemical markers were analyzed at the UHCW certified biochemistry laboratories. Normal ranges are given for TSH as 0.27–4.2 mIU/L, free-T4 (fT4) as 9–26 pmol/L, and free-T3 (fT3) as 2.8–7.1 pmol/L. Circulating levels of reverse-T3 (rT3) were measured by a competitive inhibition enzyme immunoassay [Caltag-Medsystems Ltd; inter- and intra-assay coefficients of variation (CV): 12 and 10%, respectively]. Serum leptin was measured using ELISA (human leptin Quantikine ELISA, Bio-Techne; inter- and intra-assay CV: 5.4 and 3.3%, respectively), according to the manufacturer's protocol.

### Thyroid ultrasound (US) and gray scale measurements

US examinations included gray-scale histogram analyses, as previously described (13). All thyroid US examinations were performed by the same two Consultant Radiologists with extensive experience in thyroid imaging, using a 7.5 MHz transducer (GE Healthcare Medical Systems US, Logic 8 & E9, USA). Echogenicity was assessed using sagittal images from the right thyroid lobe. The respective regions of interest (ROI) were chosen in an area in the upper right lobe with control in nearby neck muscle. Chosen ROIs were verified by an independent Radiologist. Thyroid volume was calculated as the respective largest diameters (length ^*^ width ^*^ depth) ^*^0.479 of the left lobe, the right lobe and the isthmus. The mean echogenicity (density) of the thyroid parenchyma was expressed in gray-scales relative to the control muscle echogenicity, as determined on the US gray-scale histogram analysis.

### Statistical analysis

Data are presented as means ± standard error (SEM). Normality assumptions were assessed by P-P plots. Paired sample *t*-tests or Wilcoxon Sign Rank tests were used to compare baseline and longitudinal parameters, as appropriate. Correlations between continuous variables with and without normal distribution were tested by Pearson r or Spearman's rho coefficients, respectively. *P*-values are based on two-sided tests. Significance was set at *p* < 0.05. Data analyses were performed using IBM SPSS v.22 (Chicago, Illinois, USA).

## Results

A total of 10 euthyroid, Caucasian adults with severe obesity met the study inclusion/exclusion criteria and successfully completed this study (women/men: 8/2; age: 48.6 ± 3.1 years). Of these participants, five underwent a laparoscopic adjustable gastric band procedure, while one sleeve gastrectomy and four a Roux-en-Y gastric bypass (RYGB). All bariatric procedures were performed by experienced bariatric surgeons, using standard techniques. No complications occurred in the study patients. Mean duration between baseline assessment and post-surgery follow-up was 16.5 ± 2.5 months (mean weight loss: 30.1 ± 6.0 kg).

**Figure 1:**
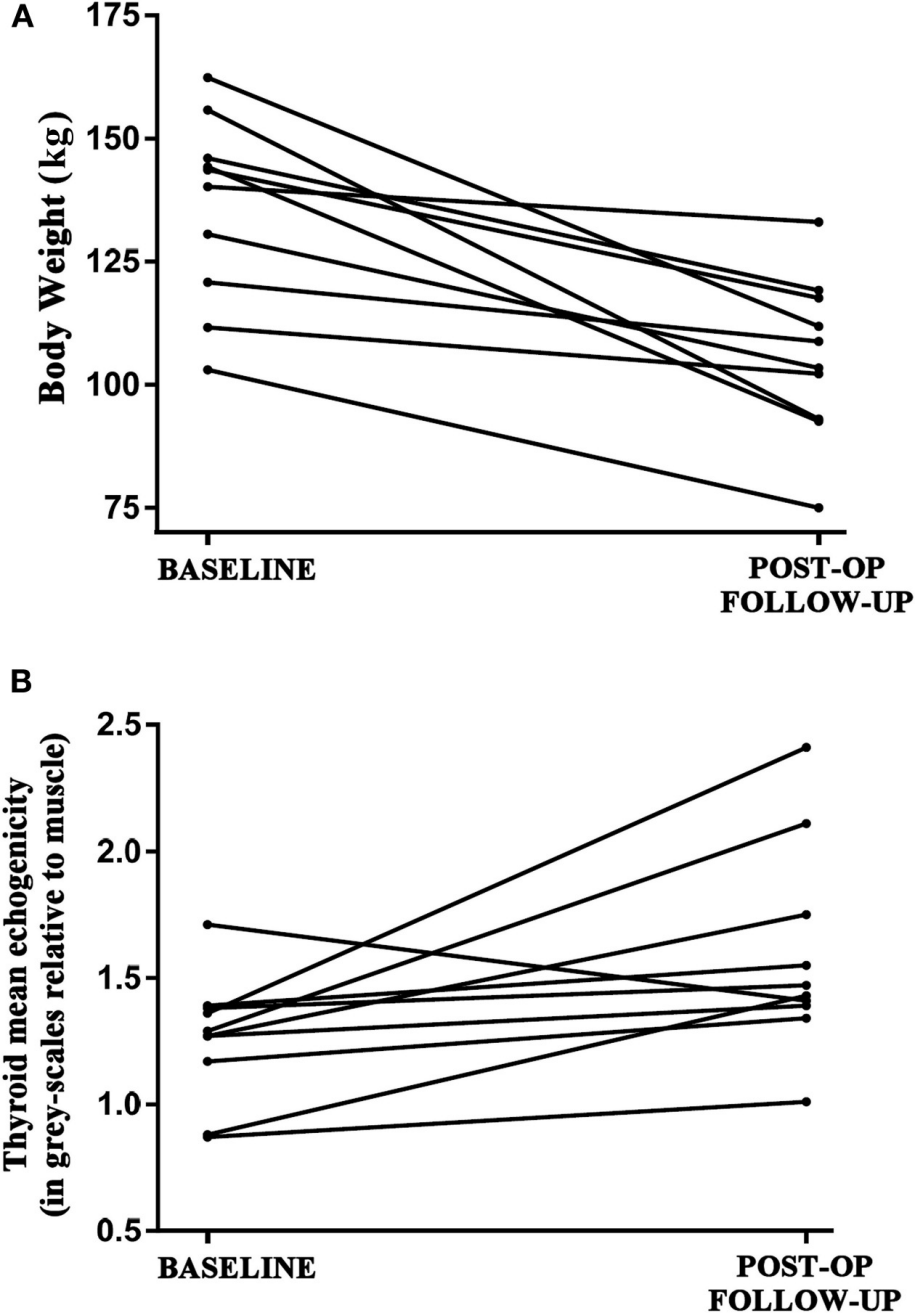
**(A)** Decrease in the absolute body weight (kg) of the euthyroid study patients (*n* = 10) between the baseline (pre- bariatric surgery) and follow-up (post bariatric surgery; after achieving at least 5% weight loss; mean duration between visits 16.5 ± 2.5 months) assessments. **(B)** Respective increase in the echogenicity of the thyroid parenchyma in the study patients between the baseline and post-surgery follow-up assessments, as expressed in gray-scales relative to the muscle echogenicity from the performed ultrasound scanning gray-scale histogram analysis.

Table 1 presents the changes in the study-pertinent characteristics of the participants between the baseline and post-surgery follow-up assessments, with significant changes in body weight, BMI, waist circumference and serum leptin levels, as expected. In parallel to the post-surgical weight loss, a significant increase was detected in the echogenicity of the thyroid parenchyma in these euthyroid patients, as expressed in gray-scales relative to the muscle echogenicity from the US gray-scale histogram analysis (Table 1, Figure 1). Thyroid volume did not change following bariatric surgery, and there were no significant changes in thyroid function tests (Table 1). Representative thyroid ultrasound scanning images before and after bariatric surgery for a study participant who achieved 18% body weight loss following bariatric surgery is presented in Supplementary Figure S1.

**Table 1:** Changes in key relevant characteristics of the study patients (*n* = 10) between the baseline and post-surgery follow-up (after at least 5% weight loss was achieved) assessments.

**Variable**	**Baseline**	**Post-surgery follow-up**	***P*-value**
Body weight (kg)	135.8 ± 6.0	105.7 ± 5.2^**^	0.001
BMI (kg/m^2^)	51.4 ± 1.8	40.0 ± 1.7^**^	0.001
WC (cm)	131.7 ± 6.4	111.0 ± 2.4^**^	0.006
Leptin (μg/L)	88.3 ± 13.9	41.6 ± 11.1^**^	0.001
TSH (mIU/L)	2.2 ± 0.3	1.5 ± 0.3	0.16
fT4 (pmol/L)	15.1 ± 0.8	14.8 ± 1.0	0.97
fT3 (pmol/L)	4.9 ± 0.3	4.7 ± 0.2	0.67
rT3 (pg/ml)	175.6 ± 31.8	196.2 ± 26.1	0.52
Thyroid echogenicity^a^	1.26 ± 0.1	1.58 ± 0.1^*^	0.03
Thyroid volume (mL)	9.3 ± 1.1	9.4 ± 0.8	0.92

*Data are presented as mean values ± standard error of the mean (SEM)*.

* P < 0.05 and

** *P < 0.01 for the comparison between the baseline and post-surgery follow-up mean values by paired sample t-test or Wilcoxon signed-rank test*.

a *Mean echogenicity (density) of the thyroid parenchyma in gray-scales relative to the muscle echogenicity, as determined by the gray-scale histogram analysis on the thyroid ultrasound scanning*.

*BMI, body mass index; WC, waist circumference; TSH, thyroid-stimulating hormone; fT4, free-T4; fT3, free-T3; rT3, reverse-T3*.

Finally, at baseline before bariatric surgery there was a statistically significant negative correlation between the mean gray scale measurements and BMI (*r* = −0.64, *p* = 0.045), but not with thyroid volume (*p* = 0.12), thyroid function tests or leptin (all *p* >0.15). The observed negative correlation of the mean gray scale measurements at baseline disappeared after bariatric-induced weight loss (*r* = 0.11, *p* = 0.77). Relative to baseline comparisons showed no significant correlation of the mean greyscale measurements with BMI (*r* = −0.26, *p* = 0.48). Overall, no significant correlations were observed between the detected increase in thyroid echogenicity and changes in anthropometric parameters and circulating leptin levels.

## Discussion

Our study presents novel, prospective data showing that in euthyroid adults with severe obesity, clinically significant weight loss achieved by bariatric surgery is associated with a significant increase in the echogenicity of the thyroid gland, as assessed on thyroid US. This finding is supported by previous studies which have shown that patients with obesity may exhibit hypo-echogenicity of the thyroid parenchyma (7, 8). Indeed, Radetti et al. have shown that overweight/obese children may frequently present with a hypo-echoic thyroid US pattern suggestive of AIT, in the absence of positive thyroid auto-antibodies (7). Moreover, in adults, Rotondi et al. have documented an unexplained hypo-echoic thyroid US pattern (in the absence of justifying thyroid disorders and negative thyroid auto-antibodies), which was present in 64.8% of the tested patients with severe obesity but only in 1.9% of the non-obese controls (8). As such, Rotondi et al. concluded that severe obesity may be associated with a hypo-echoic thyroid US pattern which can decrease the accuracy of the thyroid US for diagnosing AIT in patients with severe obesity (8). Our findings expand on these observations by showing a 25% significant increase of thyroid echogenicity on US following bariatric-induced weight loss, thus suggesting that obesity-related morphological changes of the thyroid can be reversible with weight loss.

Fat accumulation in the thyroid of patients with severe obesity has been previously hypothesized as a potential explanation for thyroid morphological changes (7, 8). However, on US, fat tissue accumulation would be expected to be hyper-echoic, i.e.,result in higher, rather than lower gray-scale readings. Therefore, in agreement with previous studies (7, 8), we also suggest that the hypo-echoic thyroid US pattern observed in severely obese patients might be attributed to the presence of obesity-related low-grade inflammation (and thus, potential infiltration of various tissues, including the thyroid, with water-containing, low-echoic pro-inflammatory cells), which is a key underlying mechanism in the pathophysiology of obesity (2). The observed increase in the thyroid US echogenicity following bariatric-induced weight loss in our study is supporting this hypothesis, although further studies including the sampling of thyroid tissue biopsies are desirable to prove this concept.

Thyroid function tests did not significantly change in the here investigated patients following bariatric surgery-induced weight loss. In contrast, a retrospective cohort analysis of 55 euthyroid patients with severe obesity has previously reported that weight loss after RYGB significantly increases circulating fT4 levels, without affecting TSH (14). More recently, a large retrospective observational study in 949 euthyroid patients with severe obesity showed that bariatric surgery (laparoscopic adjustable gastric band, sleeve gastrectomy or RYGB) can result in a decrease in TSH levels, which is independently associated with the post-surgery excess body weight loss and is greater in patients with high-normal TSH (15). Overall, a recent meta-analysis on the impact of bariatric surgery on the thyroid function in patients with obesity has shown that decreased TSH, T3, and fT3 levels can be expected in these patients post-op, without significant changes in T4, fT4, and rT3 (12). The small sample size of our bariatric cohort and the inclusion of only euthyroid adults may account for the lack of significant changes in the thyroid hormones measured in our study, although a trend to improved TSH levels could be stated.

Finally, it is noteworthy that the underlying mechanisms mediating thyroid function changes in obesity are not fully clarified yet (3–6). Notably, leptin appears involved in these mechanisms, since this pleiotropic adipokine, which regulates satiety and body weight, has also been implicated in the regulation of the thyroid axis/function in humans (3–6, 16–18). In our study, there was no correlation between the noted increase in the thyroid echogenicity and the decrease in circulating leptin after the post-surgery weight loss. Large prospective studies are needed to elucidate the exact effects of bariatric-induced weight loss on the thyroid function of patients with obesity; and their potential underlying associations to key adipokines, such as leptin.

### Study limitations

The small sample size of our cohort is a limitation. However, this pilot study was performed to guide future larger studies in this field and, indeed, was able to address our primary objective by detecting significant changes in the echogenicity of the thyroid parenchyma following bariatric-induced weight loss. Lack of a control group including patients with obesity that have not undergone bariatric surgery may be also considered as a study limitation. Moreover, due to the ethics approved design/protocol of the present pilot study, we were not able to further study the histopathological basis of the observed thyroid US imaging findings [e.g., by obtaining fine-needle aspiration cytology (FNAC) samples of the thyroid tissue or examining thyroid tissue samples following total or hemi-thyroidectomy]. However, it should be also noted that thyroid FNAC is not regarded as an appropriate technique method for elucidating the histopathological basis of such thyroid US findings, particularly for assessing the thyroid fat content (7, 8). Finally, all study participants were Caucasians, thus our findings cannot be directly extrapolated to subjects of other ethnic backgrounds.

### Conclusion

To the best of our knowledge, this is the first study in euthyroid adults with severe obesity showing that marked weight loss achieved by bariatric surgery is followed by a parallel significant increase in the thyroid US echogenicity. This novel observation expands on the existing evidence regarding changes of the thyroid morphology in obesity and is relevant to clinical practice, given that thyroid US is the most commonly used non-invasive tool for diagnosing thyroid disorders. Further, the number of patients treated with bariatric surgery is expected to keep increasing due to the ongoing obesity epidemic. As such, clinicians managing such patients should be aware that during the pre- and post-surgery follow-up it is possible to detect the presence of a hypo-echoic pattern on thyroid US that is not explained by thyroid autoimmunity and can subside with weight loss. Indeed, this may have implications for the diagnostic accuracy of thyroid US for AIT in patients with severe obesity pre- and post-bariatric surgery. The findings of this pilot study should be confirmed in future studies with large bariatric cohorts, which may also explore whether different bariatric operations induce differential effects on the thyroid morphology.

## Author contributions

MW and IK were responsible for the conception and design of the study, performed data analyses, and wrote the first and final drafts of the manuscript. OA and NH performed the US and gray scale measurements. SW and AW performed the gray scale analyses. DG and CT were responsible for the biochemical analyses. SS, LP, TB, VM, HR, and MT supported the recruitment of the participants, organization of the study and/or data collection. All authors contributed to manuscript revision and approved the final version of the manuscript.

### Conflict of interest statement

The authors declare that the research was conducted in the absence of any commercial or financial relationships that could be construed as a potential conflict of interest. The reviewer GE and handling Editor declared their shared affiliation.

## Supplementary material

Supplementary Figure S1 Representative thyroid ultrasound scanning images before **(A)** and after **(B)** bariatric surgery for a study participant who achieved 18% body weight loss following bariatric surgery.

## Abbreviations

AIT: autoimmune thyroiditis
fT4: free-T4
fT3: free-T3
rT3: reverse-T3
ROI: regions of interest
RYGB: Roux-en-Y gastric bypass
TPO-Abs: thyroid peroxidase antibodies
UHCW: University Hospitals Coventry and Warwickshire NHS Trust.
