# Supplementary figures and images for: Improved Thyroid Hypoechogenicity Following Bariatric-Induced Weight Loss in Euthyroid Adults With Severe Obesity—a Pilot Study
Source: Front Endocrinol (Lausanne). 2018 Aug 24;9:488. doi: 10.3389/fendo.2018.00488 (PMC6117911; doi:10.3389/fendo.2018.00488)

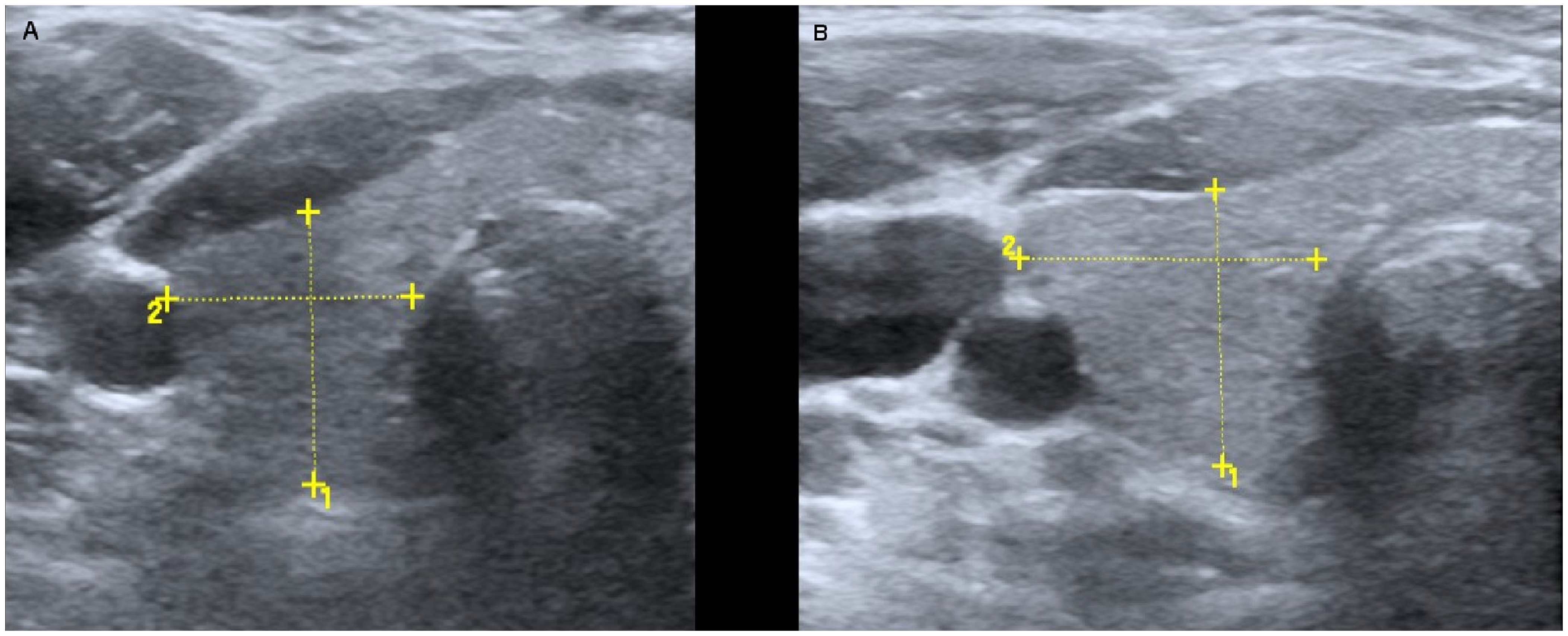

Supplement: Supplementary Figure S1 — Representative thyroid ultrasound scanning images before (A) and after (B) bariatric surgery for a study participant who achieved 18% body weight loss following bariatric surgery. [file Image_1.tiff]
